# Correction: Lovastatin fails to improve motor performance and survival in *methyl-CpG-binding protein2*-null mice

**DOI:** 10.7554/eLife.38909

**Published:** 2018-06-29

**Authors:** Claudia Villani, Giuseppina Sacchetti, Renzo Bagnati, Alice Passoni, Federica Fusco, Mirjana Carli, Roberto William Invernizzi

Villani C, Sacchetti G, Bagnati R, Passoni A, Fusco F, Carli M, Invernizzi RW. 2016. Lovastatin fails to improve motor performance and survival in methyl-CpG-binding protein2-null mice. *eLife* **5**:e22409. doi: 10.7554/eLife.22409.

Published 28, November 2016

Recently, a reader noticed that concentration units for brain levels of 24S-OHC in the legend to Table 2 were not correct. We discovered that in preparing Table 2 and Table 2—source data 1, we inadvertently assigned the wrong units to 24S-OHC concentration (ng/g instead of ng/mg).

Below, the original text of Table 2 legend:

“Table 2. Effect of Mecp2 deletion on brain levels of 24S-OHC.

Brain levels of 24S-OHC, expressed as ng/g of tissue, were measured in 28 (PND28) and 56 (PND56) day old *Mecp2*-null and WT male mice and in PND177 *Mecp2*^+/-^ females and WT mice. Food was removed from cage 6 hr before sacrifice. Data are means ± SEM of 5–6 mice per group. **p<0.01, ***p<0.001 vs. WT (Student’s t-test)”

and the corrected version:

“Table 2. Effect of Mecp2 deletion on brain levels of 24S-OHC.

Brain levels of 24S-OHC, expressed as ng/mg of tissue, were measured in 28 (PND28) and 56 (PND56) day old *Mecp2*-null and WT male mice and in PND177 *Mecp2*^+/-^ females and WT mice. Food was removed from cage 6 hr before sacrifice. Data are means ± SEM of 5–6 mice per group. **p<0.01, ***p<0.001 vs. WT (Student’s t-test)”

Concentration units in Table 2—Source data 1 have been corrected accordingly. The correction does not change any of the conclusions that we drew from these data. We apologize for any confusions this oversight may have caused.

## Additional files

Supplementary file 1. The corrected version of Table 2—source data 1 from article 22409.

