# Supplementary material for: Lovastatin fails to improve motor performance and survival in methyl-CpG-binding protein2-null mice
Source: eLife. 2016 Nov 28;5:e22409. doi: 10.7554/eLife.22409 (PMC5132339; doi:10.7554/eLife.22409)
Supplement: Supplementary file 1. — DOI: http://dx.doi.org/10.7554/eLife.22409.016 [file elife-22409-supp1.docx]

**Supplementary file 1**

**Instrumental conditions for the HPLC-MS analysis of hydroxysterols (RT, retention time; IS, internal standard used; CE, collision energy)**

| **Compound** | **RT (min)** | **IS** | **Molecular formula** | **Parent ion [M+NH4]^+^m/z** | **Product 1** | | **Product 2** | |
| --- | --- | --- | --- | --- | --- | --- | --- | --- |
|  |  |  |  |  | **m/z** | **CE (eV)** | **m/z** | **CE (eV)** |
| 22(R)-hydroxycholesterol | 16.6 | (a) | C_27_H_46_O_2_ | 420.4 | 367.3 | 18 | 161.1 | 31 |
| 22(S)-hydroxycholesterol | 21.9 | (a) | C_27_H_46_O_2_ | 420.4 | 367.3 | 18 | 161.1 | 31 |
| 24(R)-hydroxycholesterol | 19.8 | (a) | C_27_H_46_O_2_ | 420.4 | 367.3 | 18 | 283.2 | 21 |
| 24(S)-hydroxycholesterol | 19.1 | (a) | C_27_H_46_O_2_ | 420.4 | 367.3 | 18 | 283.2 | 21 |
| 25-hydroxycholesterol | 19.4 | (b) | C_27_H_46_O_2_ | 420.4 | 367.3 | 18 | 147.1 | 40 |
| 25(R)-27-hydroxycholesterol | 20.9 | (b) | C_27_H_46_O_2_ | 420.4 | 367.3 | 18 | 147.1 | 40 |
| 25(S)-27-hydroxycholesterol | 20.9 | (b) | C_27_H_46_O_2_ | 420.4 | 367.3 | 18 | 147.1 | 40 |
| 24(R,S)-hydroxycholesterol-D7 (a) | 19.0-19.7 |  | C_27_H_39_D_7_O_2_ | 426.4 | 373.3 | 18 |  |  |
| 25-hydroxycholesterol-D6 (b) | 19.3 |  | C_27_H_40_D_6_O_2_ | 427.4 | 374.3 | 18 |  |  |
